# Anti-Inflammation and Anti-Melanogenic Effects of Maca Root Extracts Fermented Using *Lactobacillus* Strains

**DOI:** 10.3390/antiox12040798

**Published:** 2023-03-24

**Authors:** Jisun Yang, Hyeijin Cho, Minchan Gil, Kyung Eun Kim

**Affiliations:** 1 Department of Cosmetic Sciences, Sookmyung Women’s University, Seoul 04310, Republic of Korea; 2 Department of Health Industry, Sookmyung Women’s University, Seoul 04310, Republic of Korea

**Keywords:** maca root, *Lepidium meyenii*, postbiotics, anti-melanogenesis, anti-inflammation

## Abstract

Maca is a well-known biennial herb with various physiological properties, such as antioxidant activity and immune response regulation. In this study, the antioxidant, anti-inflammatory, and anti-melanogenic effects of fermented maca root extracts were investigated. The fermentation was carried out using *Lactobacillus* strains, such as *Lactiplantibacillus plantarum subsp. plantarum*, *Lacticaseibacillus rhamnosus*, *Lacticaseibacillus casei*, and *Lactobacillus gasseri*. In RAW 264.7 cells, the non-fermented maca root extracts increased the secretion of nitric oxide (NO), an inflammatory mediator, in a dose-dependent manner. In contrast, the fermented extracts showed considerably lower NO secretion than the non-fermented extracts at concentrations of 5% and 10%. This indicates the effective anti-inflammatory effects of fermented maca. The fermented maca root extracts also inhibited tyrosinase activity, melanin synthesis, and melanogenesis by suppressing MITF-related mechanisms. These results show that fermented maca root extracts exhibit higher anti-inflammatory and anti-melanogenesis effects than non-fermented maca root extracts. Thus, maca root extracts fermented using *Lactobacillus* strains have the potential to be used as an effective cosmeceutical raw material.

## 1. Introduction

Postbiotics, also known as ‘non-viable probiotics’ or ‘inactivated probiotics,’ are functional bioactive compounds released after bacteria are lysed or produced through the fermentation of living bacteria, which have an indirect or direct beneficial effect on host cells [1]. In contrast, probiotics are live microorganisms that affect the health of the host when administered in appropriate amounts and prebiotics are food ingredients that have beneficial effects on the gut microbiome, and include human milk oligosaccharides, lactulose, and inulin derivatives [2,3]. The impacts of probiotics on intestinal immunity and improving gastrointestinal health are well known [4,5,6]. Compared to probiotics, research on postbiotics is in its infancy. However, studies have shown that postbiotics may improve human health by strengthening the intestinal barrier, promoting antibacterial activity against intestinal pathogens, and reducing inflammation [7,8]. Evidence that these postbiotics have the potential to modulate human health points to the importance of postbiotic research [6].

Postbiotics do not contain live microorganisms, reducing the risks associated with ingestion, and confer health benefits to the host through mechanisms similar to those of probiotics [9]. Therefore, postbiotics are considered effective because compared to probiotics, they retain similar benefits but without the side effects that can occur due to the presence of living microbes [2]. In particular, in the case of cosmetics, since live microorganisms cannot be used due to safety and stability concerns, postbiotics are used as cosmetic ingredients. Several studies have shown that postbiotics have a positive effect on skin function. Indeed, the topical application of postbiotics, such as *Lactobacillus*, *Bifidobacterium*, and *Vitrescilla filiformis* microbial lysates, may provide benefits to the skin [10,11,12]. The application of *Bifidobacterium longum sp*. extract, a probiotic lysate, improved skin sensitivity and increased skin resistance to chemical and physical aggression, whereas *V. filiformis* lysate promoted the restoration of a healthy skin microbiome by reducing *Staphylococcus aureus*’s colonization of the skin, and improved skin barrier function, thus improving transepidermal water loss (TEWL) [13,14]. Additionally, a probiotic-derived ingredient (CLS02021)—a postbiotic mixture of metabolites including enzymes, organic acids, and peptides resulting from the co-fermentation of three probiotic strains—has contributed to increasing the elasticity, moisture, pore-size reduction, and wrinkle depth of the skin. Similarly, heat-killed *Lactococcus chungagnensis* CAU 1447 has shown beneficial effects on wound healing by inducing the expression of wound-healing-promoting cytokines, chemokines, and growth factors [15,16]. This suggests that the topical application of postbiotics has beneficial effects on the skin; consequently, there is a growing market trend for the inclusion of postbiotics in cosmetic ingredients and/or products [17]. In fact, several postbiotics are already widely used in cosmetics [18].

The most commonly used probiotic strains for the development of postbiotics belong to the *Lactobacillus* and *Bifidobacterium* genera [16]. Postbiotics developed using *Lactobacilli* or *Bifidobacterium* have shown the potential for immunoregulation [19,20,21]. Postbiotics using *Lactobacilli* also have shown anti-senescence potential, and antioxidant, anti-inflammation, and anti-biofilm activity [22,23,24,25]. Most of the probiotics used in the development of postbiotic products in the cosmetic market also include *Lactobacillus*, *Bifidobacterium*, and *Saccharomyces*, and these products provide functions such as skin conditioning, skin smoothing, skin regeneration, anti-inflammation, anti-wrinkling, etc. [16]. Among these, strains belonging to *Lactobacilli* are the most commonly used [26]. *Lactobacilli* include lactic acid bacteria (LAB), which are non-spore-forming, Gam-positive, and micro-aerobic bacteria that produce lactic acid, the main product of carbohydrate fermentation. The use of LAB improves the regulation of the skin’s immune system and maintains skin homeostasis [27,28,29]. *Lactobacilli* contribute to wound healing, resistance to infection by interfering with pathogens, and defense against inflammatory processes affecting the skin [30,31,32,33].

Maca (*Lepidium meyenii*) is a biennial herbaceous plant belonging to the Brassicaceae family that includes cabbage, cauliflower, and garden cress. Maca root is native to the Andes, has been cultivated for at least 2000 years, and grows at various altitudes ranging from 2800 to 5000 m above sea level [34]. It adapts well to harsh high-altitude conditions with low temperatures, strong ultraviolet (UV) rays, low oxygen levels, and varied climates [35]. Moreover, polysaccharides from maca exhibit high antioxidant activity [36,37]. In particular, macamide and macaene fractions isolated from maca have shown antioxidant activity when evaluated through DPPH (2,2-diphenyl-1-picrylhydrazyl) and ABST ((2,2′-azino-bis(3-ethylbenzothiazoline-6-sulfonic acid)) radical scavenging [38]. Maca effectively controls sexual dysfunction, memory enhancement, skin protection, and also has neuroprotective, antidepressant, and anticancer properties [34,39,40,41]. In addition, polysaccharides, or peptides, from maca regulate immunomodulatory mechanisms by enhancing the secretion of nitric oxide (NO), tumor necrosis factor-alpha (TNF-α), and interleukin-6 (IL-6) in macrophages [42,43]. There are few studies on the effects of maca on the skin. It has been reported to prevent and improve skin damage caused by UV rays and promote high-altitude wound healing by regulating the immune system [44,45].

This study aimed to determine the possibility of developing cosmetic materials with skin-whitening and anti-inflammatory functions using fermented maca root extract, a postbiotic created by fermenting maca root with *Lactobacillus* bacterial strains.

## 2. Materials and Methods

### 2.1. Maca Root Fermented by Lactobacillus

**Figure 1:**
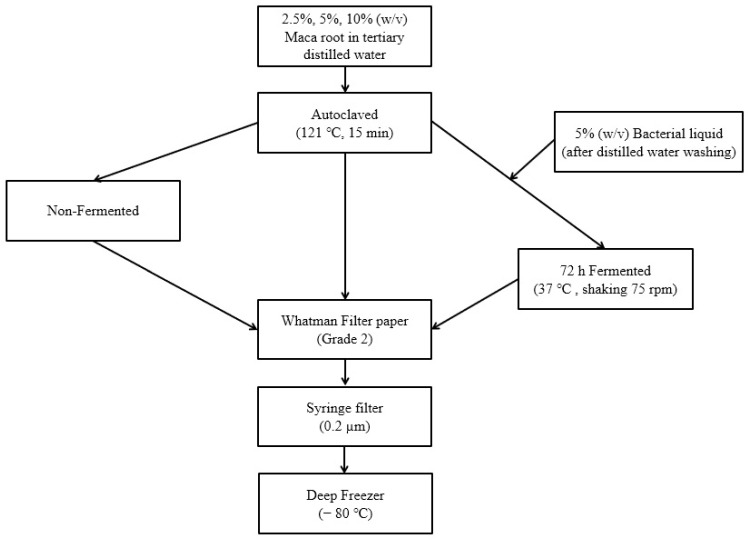
The preparation of non-fermented and fermented maca root extracts using lactic acid strains.

Maca root powder (GUAYAPI, Paris, France) was dissolved in tertiary distilled water at concentrations of 2.5, 5, and 10% (*w*/*w*). The mixture was sterilized and extracted under high pressure and temperature in an autoclave (Universal Scientific, Madison, OH, USA) at 121 °C for 15 min. *Lactiplantibacillus plantarum subsp. plantarum* (previously *Lactobacillus plantarum*) KCTC 3108, *Lacticaseibacillus rhamnosus* (previously *Lactobacillus rhamnosus*) KCTC 5033, *Lacticaseibacillus casei* (previously *Lactobacillus casei*) KCTC 3109, and *Lactobacillus gasseri* KCTC 3143 cultured in MRS (deMan Rogosa Sharpe) broth (Difco Laboratories, New York, NY, USA) were washed with tertiary distilled water and inoculated in the mixture at a concentration of 1 × 10^9^ CFU (Colony forming unit)/mL (5% *w/v*). These *Lactobacilli* were selected based on studies that suggested they would have a positive effect on skin condition. *Lactiplantibacillus plantarum subsp. plantarum* has a regulating effect on human skin health by acting against skin aging and improving skin microbiome, and *Lacticaseibacillus casei* improves skin barrier and reduces skin flakiness [46,47,48]. *Lacticaseibacillus rhamnosus* improves skin wound healing and reduces scar formation in mice, and lysates of *Lacticaseibacillus rhamnosus* improve skin barrier function in reconstructed human epidermis model [12,49]. Moreover, biosurfactants produced by *Lactobacillus gasseri* show antimicrobial properties [50]. Fermentation was performed in a shaking incubator (VISION SCIENTIFIC, Daejeon, Republic of Korea) for 72 h at 37 °C. After fermentation, the supernatant was filtered using Whatman^®^ Grade 2 qualitative filter paper (Sigma-Aldrich, St. Louis, MO, USA) and a 0.2 µm-pore-size syringe filter (HYUNDAI MICRO, Seoul, Republic of Korea) and stored at −80 °C until use (Figure 1).

### 2.2. Cell Culture

B16F10 melanoma cells were purchased from American Type Culture Collection (ATCC, Manassas, VA, USA) and RAW 264.7 cell lines were purchased from the Korean Cell Line Bank (KCBL; Seoul, Republic of Korea). Cells were maintained in high-glucose D MEM (Dulbecco Modified Eagle Medium; Cytiva Life Sciences, Logan, MA, USA) containing 1% Anti-Anti (Antibiotic-Antimycotic, Gibco, NY, USA) and 10% fetal bovine serum (FBS). The cell lines were grown in a 37 °C CO_2_ incubator (VISIONBIONEX, Bucheon, Republic of Korea) under a 5% CO_2_ atmosphere.

The lactic acid strains used for fermentation, namely, *Lactiplantibacillus plantarum subsp. plantarum* KCTC 3108, *Lacticaseibacillus rhamnosus* KCTC 5033, *Lacticaseibacillus casei* KCTC 3109, and *Lactobacillus gasseri* KCTC 3143, were purchased from Korean Collection for Type Cultures (KCTC; Seoul, Republic of Korea). These strains were cultivated in Lactobacilli MRS (deMan Rogosa Sharpe) broth (Difco Laboratories, NY, USA) at 37 °C and 75 rpm, and then sub-cultured for 48 h.

### 2.3. Cell Viability

The cytotoxicity of fermented maca root extracts on B16F10 and RAW 264.7 cells was determined using CCK-8 (Cell Counting Kit-8) solution (Donginbio, Seoul, Republic of Korea). B16F10 cells were seeded at a concentration of 2 × 10^3^/100 µL and RAW 264.7 cell lines were seeded at a concentration of 4 × 10^3^/100 µL in a culture medium in 96-well plates (SPL, New York, NY, USA). After 24 h, non-fermented and fermented maca root extracts were diluted in the culture medium by 1/100 of the total volume and treated with 100 µL in each well. After 24 h and 48 h of treatment, 20 µL of CCK-8 solution was added and incubated for 2 h at 37 °C. The absorbance of the samples was measured at 450 nm using a microplate reader (Thermo Fisher, Waltham, MA, USA). The results were the averages of triplicate samples.

$$\mathrm{Cell}\;\mathrm{viability}\;\left(\%\right)=\frac{\mathrm{Absorbance}\;450\;\mathrm{nm}\;\mathrm{of}\;\mathrm{Sample}}{\mathrm{Absorbance}\;450\;\mathrm{nm}\;\mathrm{of}\;\mathrm{Control}}\times 100$$

### 2.4. NO Assay

RAW 264.7 cells were seeded at a concentration of 3 × 10^5^ in a 6-well plate. After 24 h of seeding, 1 µg/mL lipopolysaccharide (LPS) from *Escherichia coli* (Sigma-Aldrich, St. Louis, MO, USA), or 1/100 of the total volume of non-fermented and fermented maca root extracts, was administered for treatment for 24 h. After 24 h, 100 µL of the supernatant was transferred to a 96-well plate, and 50 µL of N1 buffer from the NO Plus Detection Kit (iNtRON, Seongnam, Republic of Korea) was added and reacted at room temperature for 10 min. Then, N2 buffer was added, incubation was performed at room temperature for 10 min, and the absorbance was measured at 520 nm using a microplate reader (Thermo Fisher, Waltham, MA, USA). The amount of nitrate produced was calculated using a nitrate standard curve. The results were the averages of triplicate samples.

### 2.5. Mushroom Tyrosinase Inhibition Assay

The tyrosinase inhibition effect of the mushroom tyrosinase assay was determined based on a modified previous study [51]. Tyrosinase from mushrooms (Sigma-Aldrich, St. Louis, MO, USA) was dissolved in phosphate buffer (pH 6.6; Sigma-Aldrich, St. Louis, MO, USA) at a concentration of 7500 U/mL. L-tyrosine (Sigma-Aldrich, St. Louis, MO, USA) was used as a substrate and subsequently dissolved in 1 M HCl (hydrochloric acid) at a concentration of 15 mM. Next, 660 µL of 0.1 M sodium phosphate buffer (SPB; pH 6.8) was dispensed in a 1.5 mL Eppendorf tube and 60 µL samples were treated. Then, 90 µL of 7500 U/mL mushroom tyrosinase and 15 mM L-tyrosine were dispensed sequentially and incubated at 37 °C for 12 min. After incubation, the Eppendorf tubes were placed on ice for 1 min and 150 µL of each sample was placed in each well of a 96-well plate. Finally, absorbance was measured at 490 nm using a microplate reader (Thermo Fisher, Waltham, MA, USA). In the control, tertiary diluted water was used instead of the sample, and arbutin (500 µg/mL; Sigma-Aldrich, St. Louis, MO, USA) was used as a positive control. The results were the averages of triplicate samples.

$$\mathrm{Tyrosinase}\;\mathrm{inhibition}\;\mathrm{rate}\;\left(\%\right)=100\times (1\;-\;\frac{B\;-\;B^{\prime }}{A\;-\;A^{\prime }})$$

A: Tertiary diluted water instead of sample;

A′: Without tyrosinase in condition A;

B: Treated sample;

B’: Without tyrosinase in condition B.

### 2.6. Intracellular Melanin Content Assay

Analysis of melanin content was carried out based on modified previous reports [52]. B16F10 cells were seeded at a density of 2 × 10^5^ in a 60 mm culture dish (SPL, New York, NY, USA). After 24 h, 200 nM α-melanocyte stimulating hormone (α-MSH; Sigma-Aldrich, St. Louis, MO, USA) and 1/100 of the total volume of maca root extracts and fermented maca root extracts or arbutin (500 µg/mL) were administered for treatment for 48 h. The culture medium was removed. Next, the cells were removed using 1 mL PBS (phosphate buffered saline) and transferred to a 1.5 mL Eppendorf tube. The collected cells were centrifuged at 13,000 rpm for 10 min. After centrifugation, the supernatant was removed, and 650 µL of 1 N NaOH (Sigma-Aldrich, St. Louis, MO, USA) solution, in which 10% DMSO (Dimethyl sulfoxide; Sigma-Aldrich, St. Louis, MO, USA) was dissolved, was added to the pellet. The mixture was dissolved at 80 °C for 1 h. Then, the tubes were vortexed and 200 µL samples were transferred to a 96-well plate and the absorbance was measured at 405 nm using a microplate reader (Thermo Fisher, Waltham, MA, USA). Moreover, 500 µg/mL arbutin (Sigma-Aldrich, St. Louis, MO, USA) was used as a positive control. The results were the averages of triplicate samples.

### 2.7. Extracellular Melanin Content Assay

Extracellular melanin content was determined with reference to modified previous reports [53]. B16F10 cells were seeded at a density of 2 × 10^5^ in a 60 mm culture dish (SPL, New York, NY, USA). After 24 h, the medium was removed. Then, 200 nM α-MSH and 1/100 of the total volume of maca root extracts and fermented maca root extracts or arbutin (500 µg/mL) were administered for treatment for 48 h using DMEM without phenol red (Cytiva Life Sciences, Logan, MA, USA). After 48 h of treatment, 200 µL of the medium was transferred to a 96-well plate. Absorbance was subsequently measured at 405 nm using a microplate reader (Thermo Fisher, Waltham, MA, USA). The results were the averages of triplicate samples.

### 2.8. Intracellular Tyrosinase Activity

Analysis of intracellular tyrosinase activity was carried out with reference to modified previous research [54]. B16F10 cells were seeded in 60 mm culture dishes at a concentration of 2 × 10^5^. After 24 h, 200 nM α-MSH and 1/100 of the total volume of non-fermented and fermented maca root extracts were administered for treatment for 48 h. Cells were collected and centrifuged at 13,000 rpm for 10 min. The supernatant was removed. Then, pellets were suspended in 100 µL phosphate buffer (pH 6.6) containing 1% (*v*/*v*) Triton X-100 (Sigma-Aldrich, St. Louis, MO, USA) and shaken on ice for 40 min. Next, the cells were centrifuged at 13,000 rpm for 20 min and the supernatant was collected for quantification. Sodium phosphate buffer (0.1 M; pH 7) was used as a solvent, and 40 µg of protein was added to a total volume of 40 µL. A total of 40 µg of protein was incubated with 200 µL of 10 mM L-DOPA (L-3,4-dihydroxyphenylalanine) in the dark at 37 °C for 30 min. After the reaction, absorbance was measured at 475 nm using a microplate reader (Thermo Fisher, Waltham, MA, USA). Arbutin (500 µg/mL) was used as a positive control. The results were the averages of triplicate samples.

$$\mathrm{Intracellular}\;\mathrm{tyrosinase}\;\mathrm{activity}\;\left(\%\right)=\frac{\mathrm{Absorbance}\;\mathrm{of}\;\mathrm{Sample}\;\mathrm{at}\;475\;\mathrm{nm}\;}{\mathrm{Absorbance}\;\mathrm{of}\;\mathrm{Control}\;\mathrm{at}\;475\;\mathrm{nm}\;}\times 100$$

### 2.9. Quantitative Real-Time PCR

There are three essential enzymes for melanin synthesis: *tyrosinase (TYR), tyrosinase-related protein 1 (TRP-1)*, and *tyrosinase related protein 2 (TRP-2)* [55]. These enzymes are transcriptionally regulated by *microphthalmia-associated transcription factor (MITF)*, a master gene for melanocyte differentiation [56]. Therefore, in this study, *MITF, tyrosinase* and *TRP-2* mRNA expression was determined. B16F10 RNA was extracted using TRIzol reagent (Thermo Fisher, Waltham, MA, USA). The extracted RNA was reverse-transcribed to synthesize cDNA using Superscript VILO (Invitrogen, Waltham, MA, USA). Next, PCR was performed using a 2× qPCRBIO SyGreen Mixture (PCRBIOSYSTEMS, Wayne, PA, USA) as well as forward and reverse primers. PCR conditions were as follows: after pre-incubation at 95 °C for 2 min, denaturation at 95 °C for 5 s and annealing at 60 °C for 30 s were repeated for 40 cycles, and extension was performed at 72 °C for 5 min. qRT-PCR was performed using a The LightCycler^®^ 96 instrument (Roche Life Science, Pleasanton, CA, USA). GAPDH was used as the housekeeping gene. The results were the averages of duplicate samples. The primers used in the experiments are listed in Table 1.

### 2.10. DPPH Antioxidant Assay

The antioxidant effects of non-fermented and fermented maca root extracts were determined using the OxiTec^TM^ DPPH antioxidant assay kit (BIOMAX, Guri, Republic of Korea) according to the manufacturer’s protocol. Non-fermented and fermented maca root extracts (2.5% and 5%, respectively) were prepared, and the assay buffer and DPPH working solution were individually added to a 96-well plate. The plate was incubated for 30 min at room temperature in the dark. Finally, absorbance was measured at 517 nm using a microplate reader. Ascorbic acid (500 µg/mL) was used as a positive control. The results were the averages of triplicate samples.

$$\mathrm{Inhibition}\;\mathrm{ratio}\;\mathrm{of}\;\mathrm{sample}\;\left(\%\right):\;(\frac{\mathrm{Acs}\;-\;\mathrm{As}}{\mathrm{Acs}})\;\times \;100$$

A_cs_: Blank 1—Blank 2;

A_s_: Abs of Sample—Blank 2.

### 2.11. Determination of Total Phenolic Content

The total phenolic content (TPC) was determined according to the method described previously with slight modifications [59]. After mixing 2N Folin-Ciocalteu’s phenol reagent (Sigma-Aldrich, St. Louis, MO, USA) and distilled water at a ratio of 1:2, 10 µL of the mixture and 10 µL of sample were mixed and left in the dark for 3 min. Then, 150 µL of 20% Na_2_CO_3_ (Sigma-Aldrich, St. Louis, MO, USA) was added and reacted in the dark for 1 h, and absorbance was measured at 750 nm using a microplate reader (Thermo Fisher, Waltham, MA, USA). The concentration of TPC in the samples was obtained by substituting the measured absorbance into a standard calibration curve prepared using gallic acid (Sigma-Aldrich, St. Louis, MO, USA) as a standard material. The results were the averages of triplicate samples.

### 2.12. Statistical Analysis

All data were analyzed using the GraphPad Prism software (version 8.0). Statistical significance was assessed using unpaired two-tailed Student’s *t*-tests. *p* < 0.05 was considered statistically significant (* *p* < 0.05, ** *p* < 0.005, *** *p* < 0.0005).

## 3. Results

### 3.1. Effects of Antioxidants of Non-Fermented Maca Root Extracts Maintained through Fermentation by Lactobacilli

**Figure 2:**
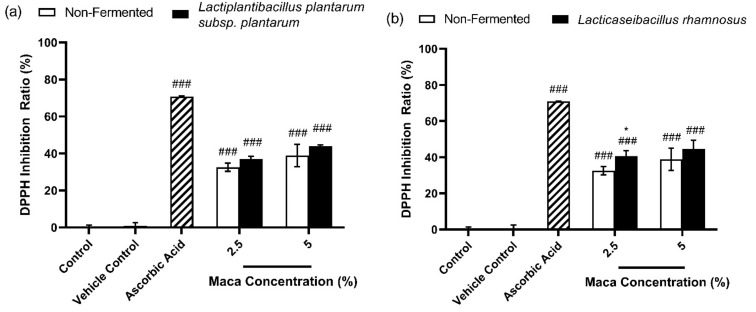

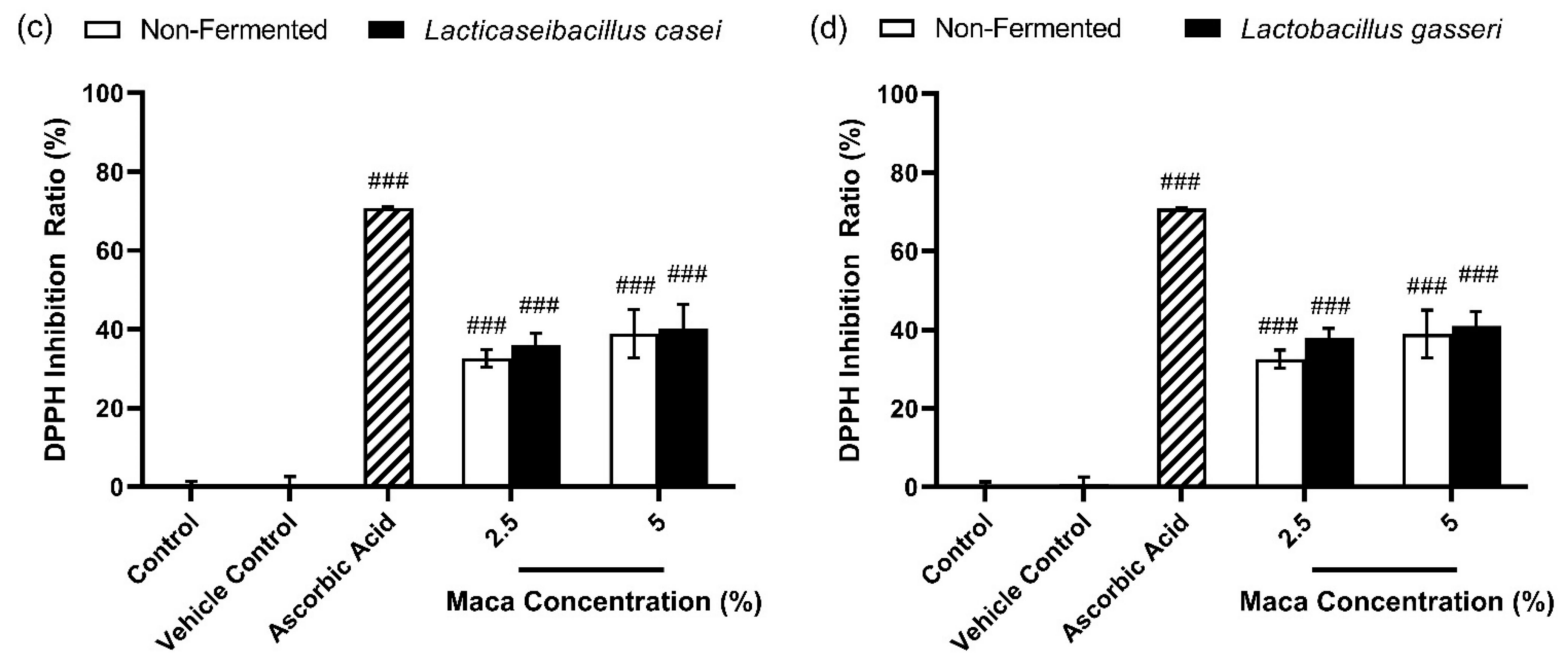
Effects of non-fermented and fermented maca root extracts on antioxidant activity evaluated using the DPPH assay. Treated with maca root extracts fermented using (**a**) *Lactiplantibacillus plantarum subsp. plantarum* KCTC 3108, (**b**) *Lacticaseibacillus rhamnosus* KCTC 5033, (**c**) *Lacticaseibacillus casei* KCTC 3109, and (**d**) *Lactobacillus gasseri* KCTC 3143. Error bars, mean ± SD. ### *p* < 0.0005 compared with the vehicle control group and * *p* < 0.05 compared to the same concentration of non-fermented maca root extracts.

The antioxidant effects of 2.5% and 5% of the non-fermented and fermented maca root extracts are shown in Figure 2. Here, 2.5% and 5% concentrations of the non-fermented maca root extracts showed approximately 32% and 39% DPPH inhibition ratios, respectively. At a concentration of 2.5%, the maca root extracts fermented using *Lactiplantibacillus plantarum subsp. plantarum* KCTC 3108, *Lacticaseibacillus rhamnosus* KCTC 5033, *Lacticaseibacillus casei* KCTC 3109, and *Lactobacillus gasseri* KCTC 3143 showed DPPH inhibition ratios of approximately 36.9%, 40.6%, 36.1%, and 37.9%, respectively. Similarly, the DPPH inhibition ratios were approximately 43.9%, 44.6%, 40.2%, and 40.9%, respectively, at a concentration of 5%. This suggests that the fermentation of maca root extracts maintains the antioxidative effect of this plant.

### 3.2. Total Phenolic Content of Non-Fermented Maca Root Extracts Maintained through Fermentation by Lactobacilli

It is well known that plant phenolic compounds form one of the major groups of compounds that act as major antioxidants or free radical scavengers, and whole plant polyphenols are effective as singlet-reducing agents, oxygen scavengers, and hydrogen atom donators [60]. Therefore, the total phenolic content (TPC) of the non-fermented and fermented maca root extracts were measured for each concentration. The content of phenolic compounds was calculated using calibration curve, expressed in gallic acid equivalents (GAE) as milligrams per 100 µL of the extracts (mg GAE/100 µL extracts). The TPC of the non-fermented maca root extracts was approximately 3.06, 4.91, and 9.19 mg GAE/100 µL extracts at concentration of 2.5, 5, and 10%, respectively (Table 2). The fermented maca root extracts using *Lactiplantibacillus plantarum subsp. plantarum* KCTC 3108, *Lacticaseibacillus rhamnosus* KCTC 5033, *Lacticaseibacillus casei* KCTC 3109, and *Lactobacillus gasseri* KCTC 3143 showed no significant difference in TPC compared to the non-fermented group at concentrations of 2.5, 5, and 10%.

### 3.3. Fermented Maca Root Extracts Suppresses NO Production Compared to Non-Fermented Maca Root Extracts

Some maca root components may play an immunoregulatory role by releasing pro-inflammatory cytokines and producing NO [42,43]. However, these pro-inflammatory cytokines contribute to the exacerbation of allergic asthma [61]. In addition, NO is a highly reactive molecule/free radical that can have oxidative properties [62]. Therefore, the NO production must be suppressed to be developed as a cosmetic ingredient. In this study, the NO concentration was measured to determine whether the inflammatory response was suppressed during the maca root fermentation.

**Figure 3:**
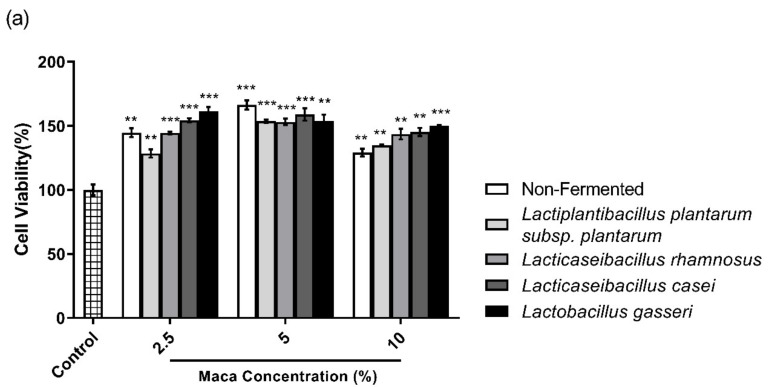

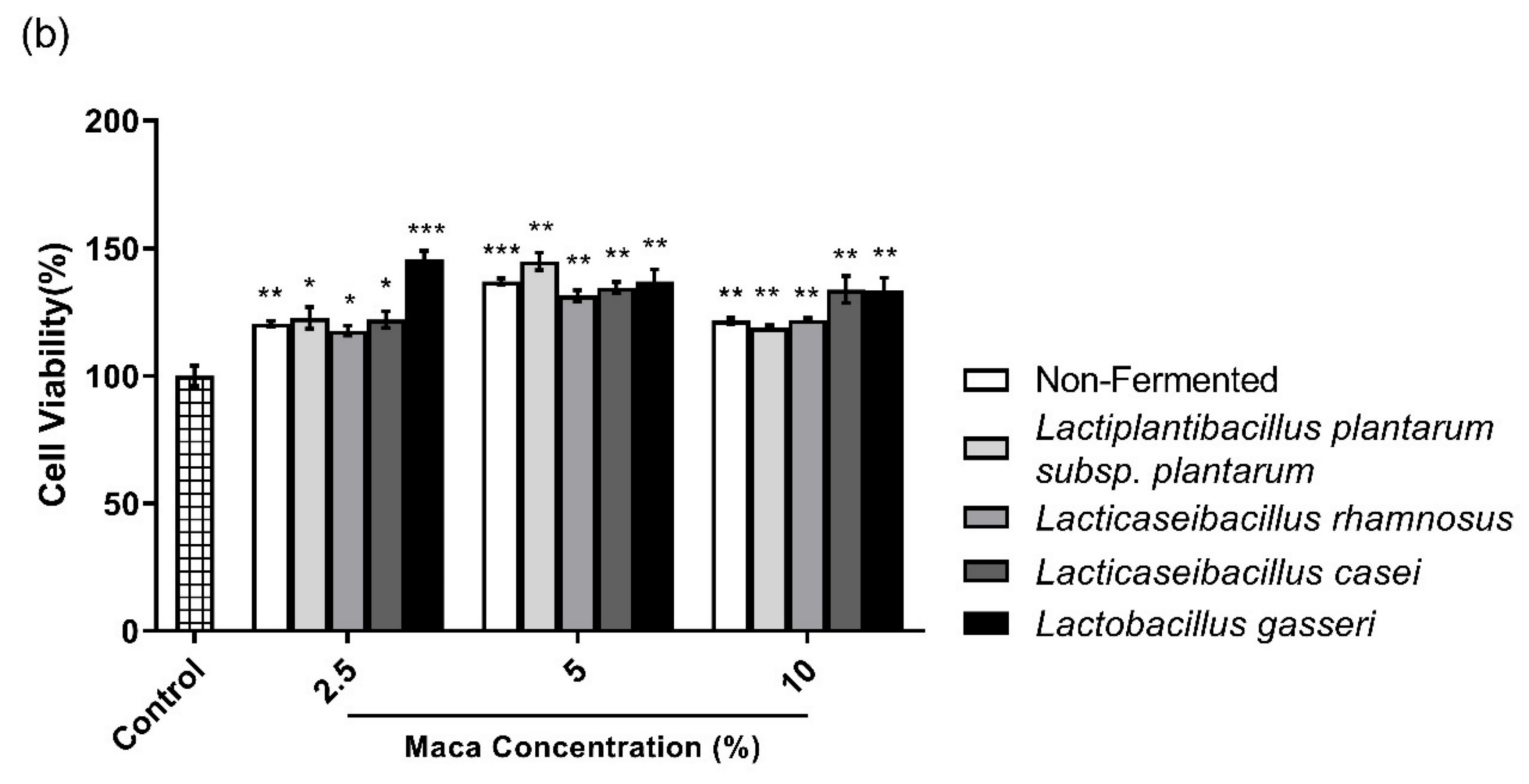
Cell viability of non-fermented and fermented maca root extracts on the RAW 264.7 cell line. Non-fermented and fermented maca root extracts’ effects on the cell viability of RAW 264.7 cells were examined for (**a**) 24 h and (**b**) 48 h using CCK-8 reagent. The results shown are the averages of triplicate samples. Error bars, mean ± SD. * *p* < 0.05, ** *p* < 0.005, *** *p* < 0.0005 compared to the control group.

To confirm the concentration without cytotoxicity in RAW 264.7, cell viability was first measured for each concentration of the non-fermented and fermented maca root extracts using CCK-8 reagent. The non-fermented and fermented maca root extracts were treated with 1/100 of the total medium volume for 24 h and 48 h and compared with the control group, which was treated with tertiary diluted water. Figure 3 shows the viability percentages of RAW 264.7 cells after (a) 24 h and (b) 48 h of treatment. The non-fermented and fermented maca root extracts showed over 100% cell viability compared to the control. As a result, the treatment of the non-fermented and fermented maca root extracts did not show any cytotoxicity on RAW 264.7 cells for 24 h and 48 h. Moreover, those concentrations of non-fermented and fermented maca root extracts were used to measure the NO production in RAW 264.7 cells.

**Figure 4:**
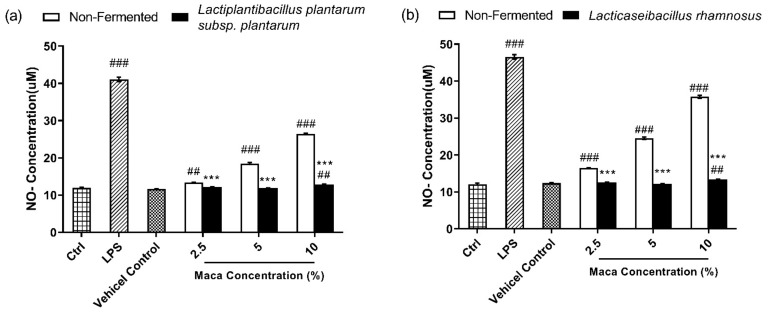

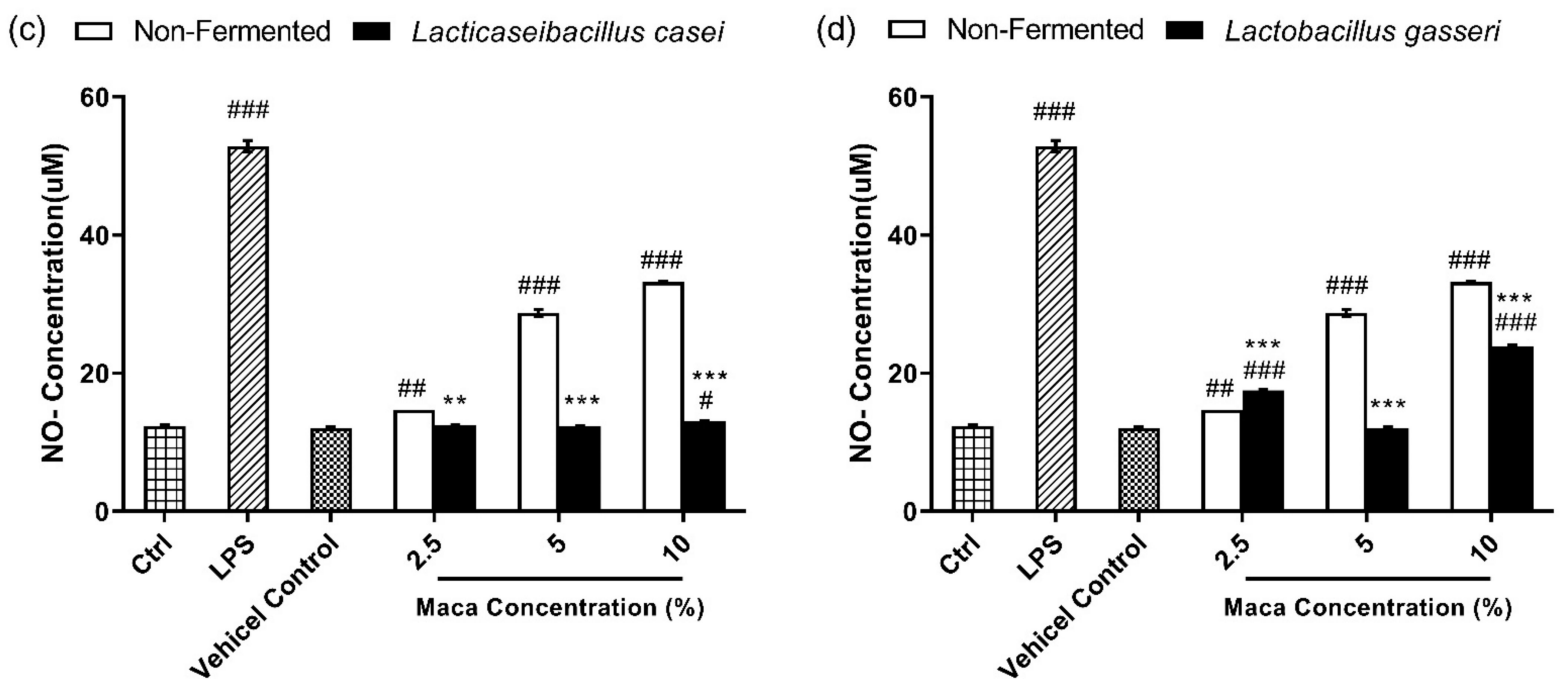
Effects of non-fermented and fermented maca root extracts on NO production in RAW 264.7 cells. NO concentrations in RAW 264.7 cells treated with maca root extracts fermented using (**a**) *Lactiplantibacillus plantarum subsp. plantarum* KCTC 3108, (**b**) *Lacticaseibacillus rhamnosus* KCTC 5033, (**c**) *Lacticaseibacillus casei* KCTC 3109, and (**d**) *Lactobacillus gasseri* KCTC 3143. Error bars, mean ± SD. # *p* < 0.05, ## *p* < 0.005, and ### *p* < 0.0005 compared to the control group, ** *p* < 0.005, *** *p* < 0.0005 compared to the same concentration of non-fermented maca root extracts.

Figure 4 compares the NO production of RAW 264.7 cells when treated with the non-fermented and fermented maca root extracts. LPS (1 µg/mL) was used as a positive control. In this study, the non-fermented maca root extracts increased the NO production in a dose-dependent manner. At 5% and 10% concentrations, the fermented maca root extracts showed an extremely low NO production compared to the non-fermented maca root extracts. This indicates that the fermented maca root extract inhibits inflammation, whereas the non-fermented maca root extract does not.

### 3.4. Fermented Maca Root Extracts Inhibit Mushroom Tyrosinase Activity

**Figure 5:**
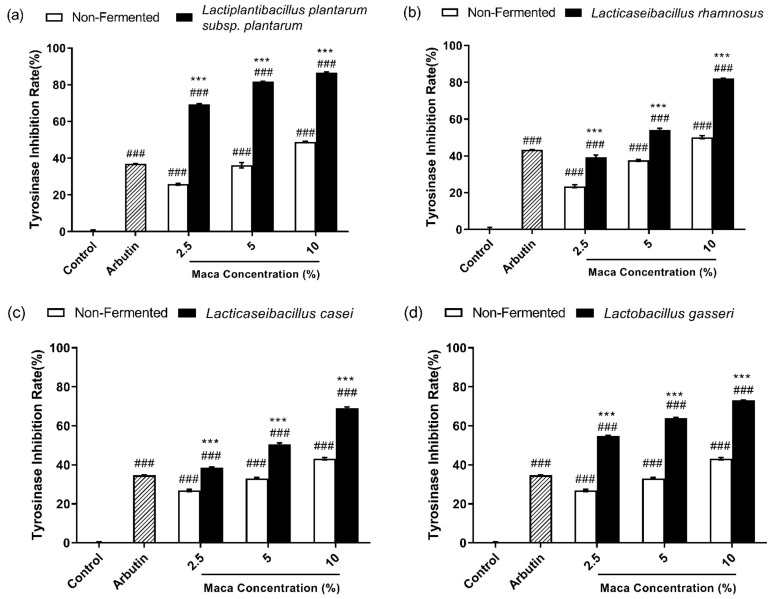
Effects of non-fermented and fermented maca root extracts on the inhibition rate (%) of mushroom tyrosinase. Treated with maca root extracts fermented using (**a**) *Lactiplantibacillus plantarum subsp. plantarum* KCTC 3108, (**b**) *Lacticaseibacillus rhamnosus* KCTC 5033, (**c**) *Lacticaseibacillus casei* KCTC 3109, and (**d**) *Lactobacillus gasseri* KCTC 3143. Error bars, mean ± SD. ### *p* < 0.0005 compared to the control group, and *** *p* < 0.0005 compared to the same concentration of non-fermented maca root extracts.

To confirm the effect of non-fermented and fermented maca roots on mushroom tyrosinase, the inhibition rate of mushroom tyrosinase activity was investigated using L-tyrosine as a substrate. Here, 500 µg/mL arbutin was used as a positive control and tertiary diluted water was added to the control group instead of the sample. As shown in Figure 5, the 2.5%, 5%, and 10% non-fermented maca root extracts showed approximately 25%, 34%, and 47% tyrosinase inhibition, respectively. These results indicate that the non-fermented maca root increased the tyrosinase inhibition rate in a dose-dependent manner. Moreover, 500 µg/mL arbutin showed approximately 35% tyrosinase inhibition. *Lactiplantibacillus plantarum subsp. plantarum* KCTC 3108 showed 69.44%, 81.72%, and 86.59% tyrosinase inhibition, respectively, in a dose-dependent manner (Figure 5a). *Lacticaseibacillus rhamnosus* KCTC 5033 showed 39.18%, 54.12%, and 82.17% tyrosinase inhibition, respectively, in a dose-dependent manner (Figure 5b). As shown in Figure 5c, *Lacticaseibacillus casei* KCTC 3109 showed tyrosinase inhibition rates of 38.5%, 50.58%, and 69.08%, respectively. Moreover, *Lactobacillus gasseri* KCTC 3143 showed 54.83%, 63.92%, and 73.17% tyrosinase inhibition rates, respectively, in a dose-dependent manner (Figure 5d). In this study, the maca root extracts exhibited tyrosinase inhibition in a dose-dependent manner, with the fermented maca root extracts showing a higher tyrosinase inhibition rate than the non-fermented maca root extracts.

### 3.5. Fermented Maca Root Extracts Suppress Melanin Synthesis

**Figure 6:**
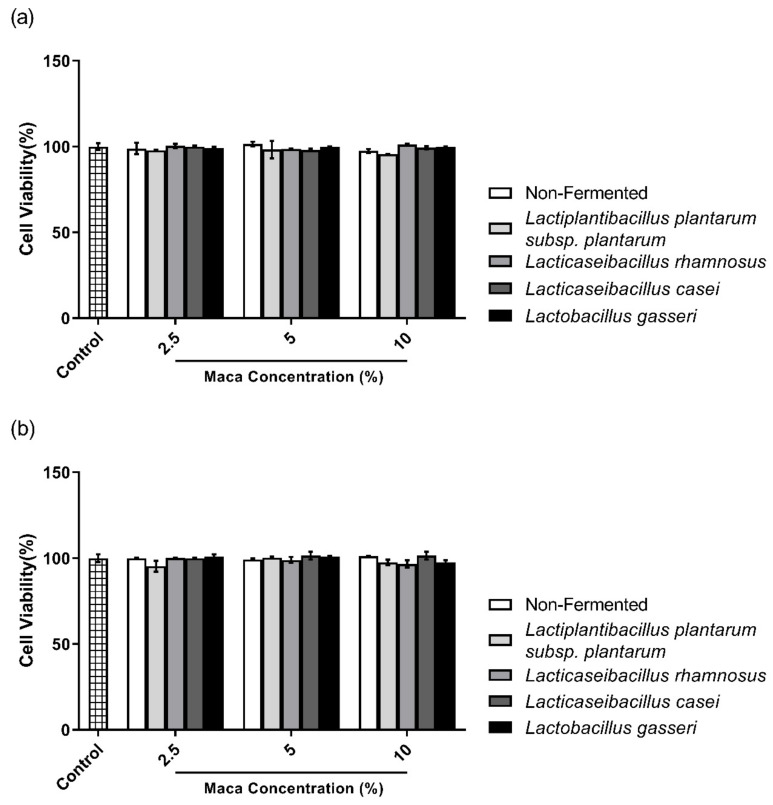
Cell viability of non-fermented and fermented maca root extracts on the B16F10 cell line. CCK-8 was used to confirm the non-fermented and fermented maca root extracts’ effects on the cell viability of B16F10 when treated for (**a**) 24 h and (**b**) 48 h.

To determine whether the non-fermented and fermented maca root extracts is cytotoxic to B16F10 melanoma cells, cell viability was confirmed using the CCK-8 assay. The non-fermented and fermented maca root extracts were treated with 1/100 of the total volume of the media for 24 h and 48 h. They were subsequently compared to the control group that was treated with tertiary diluted water instead of the sample. Figure 6 shows the viability (%) of B16F10 cells after (a) 24 h and (b) 48 h of treatment, and all samples showed more than 95% viability compared with the control. The CCK-8 assay confirmed the safety of the non-fermented and fermented maca concentrations in B16F10 cells after 24 h and 48 h of treatment.

**Figure 7:**
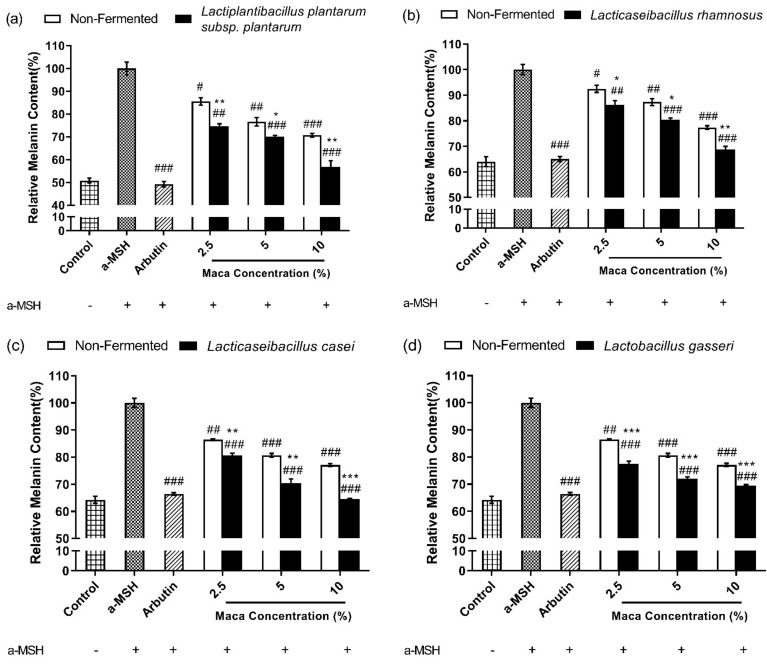
Effect of non-fermented and fermented maca root extracts on intracellular melanin content. Intracellular melanin content of B16F10 treated with maca root extracts fermented using (**a**) *Lactiplantibacillus plantarum subsp. plantarum* KCTC 3108, (**b**) *Lacticaseibacillus rhamnosus* KCTC 5033, (**c**) *Lacticaseibacillus casei* KCTC 3109, and (**d**) *Lactobacillus gasseri* KCTC 3143. Error bars, mean ± SD. # *p* < 0.05, ## *p* < 0.005 and ### *p* < 0.0005 compared to the α-MSH group, * *p* < 0.05, ** *p* < 0.005, and *** *p* < 0.0005 compared to the same concentration of non-fermented maca root extracts.

**Figure 8:**
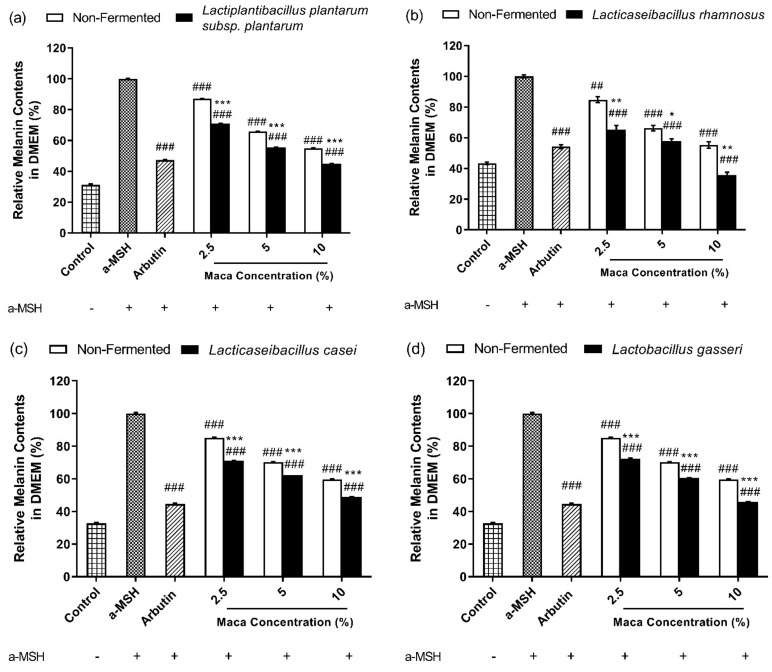
Effects of non-fermented and fermented maca root extracts on extracellular melanin content. Extracellular melanin content of B16F10 cells treated with maca root extracts fermented using (**a**) *Lactiplantibacillus plantarum subsp. plantarum* KCTC 3108, (**b**) *Lacticaseibacillus rhamnosus* KCTC 5033, (**c**) *Lacticaseibacillus casei* KCTC 3109, and (**d**) *Lactobacillus gasseri* KCTC 3143. Error bars, mean ± SD. ## *p* < 0.005 and ### *p* < 0.0005 compared to α-MSH group, * *p* < 0.05, ** *p* < 0.005, and *** *p* < 0.0005 compared to the same concentration of non-fermented maca root extracts.

As the fermented maca root extracts showed a higher tyrosinase inhibition rate in an extracellular experiment (Figure 5), we examined whether these maca root extracts influenced melanin pigment production in B16F10 cells (Figure 7). In addition, the melanin content in the medium was confirmed using DMEM without phenol red (Figure 8). The intracellular melanin content, which was treated with the non-fermented maca root extracts in a dose-dependent manner, was reduced (Figure 7). The fermented maca root extract postbiotic treatment showed a lower intracellular melanin content than the non-fermented maca root extract treatment. Figure 8 shows the relative melanin content in DMEM without phenol red after 48 h of treatment. Both the non-fermented and the fermented maca root extracts caused low extracellular melanin content in a dose-dependent manner. The fermented maca root extracts caused a lower extracellular melanin content than the non-fermented maca root extracts. These results suggest that non-fermented and fermented maca reduce both the intracellular and extracellular melanin content. Furthermore, the fermented conditions show lower melanin content than the non-fermented conditions. This indicates that the non-fermented and fermented maca root extracts reduce melanogenesis, and that the fermented maca root extracts are more effective in their anti-melanogenesis effects than the non-fermented maca root extracts.

### 3.6. Fermented Maca Root Extracts Inhibit Intracellular Tyrosinase Activity

**Figure 9:**
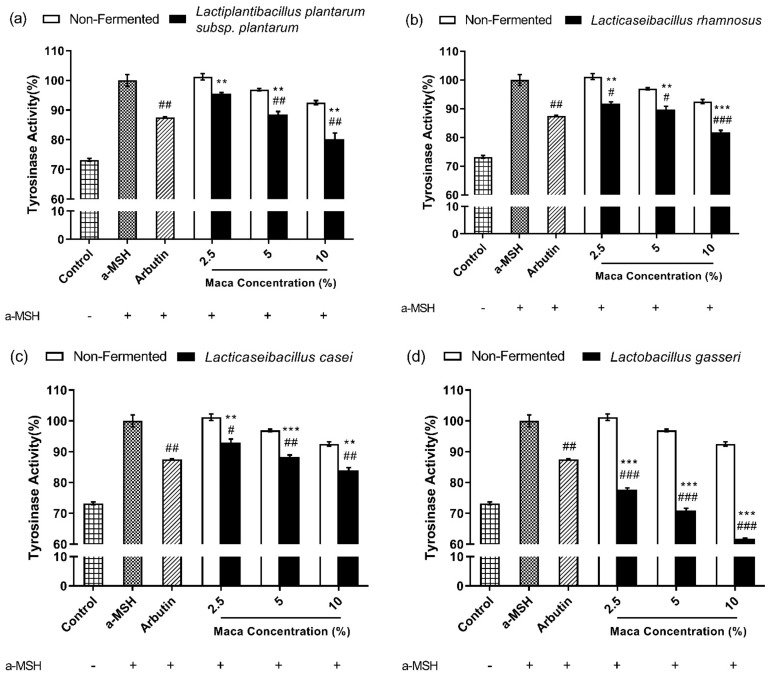
Effects of non-fermented and fermented maca root extracts on intracellular tyrosinase activity. Intracellular tyrosinase activity of B16F10 cells treated with maca root extracts fermented using (**a**) *Lactiplantibacillus plantarum subsp. plantarum* KCTC 3108, (**b**) *Lacticaseibacillus rhamnosus* KCTC 5033, (**c**) *Lacticaseibacillus casei* KCTC 3109, and (**d**) *Lactobacillus gasseri* KCTC 3143. Error bars, mean ± SD. # *p* < 0.05, ## *p* < 0.005, and ### *p* < 0.0005 compared to the α-MSH group. ** *p* < 0.005 and *** *p* < 0.0005 compared to the same concentration of the non-fermented maca root extract group.

Melanin pigments are derived from L-tyrosine, and melanin-producing enzymes, such as *tyrosinase (TYR)*, *tyrosinase-related protein 1(TRP1)* and *tyrosinase-relate d protein 2 (TRP2)*, are involved in melanogenesis. Tyrosinase is a copper-dependent enzyme that catalyzes the conversion of L-tyrosine to L-DOPA, which is a rate-limiting step in melanin synthesis [63]. As shown in Figure 5, Figure 7 and Figure 8, the non-fermented and fermented maca root extracts inhibited melanogenesis. Therefore, the inhibitory effect of the non-fermented and fermented maca root extracts on tyrosinase enzyme activity was investigated under intracellular conditions. Arbutin (500 µg/mL) was used as the positive control. The non-fermented and fermented maca root extracts inhibited tyrosinase activity under intracellular conditions in a dose-dependent manner (Figure 9). Furthermore, the fermented conditions were more effective at inhibiting intracellular tyrosinase than the non-fermented conditions. This shows that non-fermented and fermented maca root extracts affect tyrosinase activity and reduce melanin production, indicating that both types of maca root extract are possible anti-melanogenesis agents.

### 3.7. Fermented Maca Root Extracts Suppressed mRNA expression of MITF, Tyrosinase and TRP-2 in B16F10

**Figure 10:**
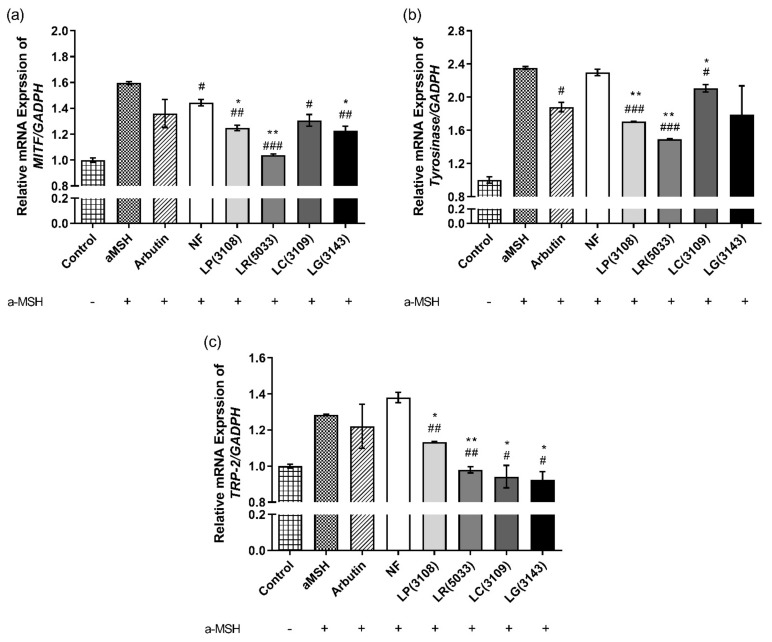
Effects of non-fermented and fermented maca root extracts on the relative mRNA expression of (**a**) MITF, (**b**) tyrosinase, and (**c**) TRP-2 in B16F10. Error bars, mean ± SD. # *p* < 0.05, ## *p* < 0.005, and ### *p* < 0.0005 compared to the α-MSH group. * *p* < 0.05 and ** *p* < 0.005 compared to the non-fermented maca root extract group. NF, non-fermented; LP(3108), *Lactiplantibacillus plantarum subsp. plantarum* KCTC 3108; LR(5033), *Lacticaseibacillus rhamnosus* KCTC 5033; LC(3109), *Lacticaseibacillus casei* KCTC 3109; LG(3143), *Lactobacillus gasseri* KCTC 3143.

The expression of the MITF, tyrosinase, and TRP-2 regulates melanogenesis by regulating the enzymatic cascade [64]. In this study, the fermented maca root extracts showed higher anti-melanogenic effects than the non-fermented maca root extracts (Figure 5, Figure 7, Figure 8 and Figure 9). To determine whether the fermented maca root extracts showed anti-melanogenic effects by regulating the expression of genes involved in melanogenesis, the mRNA expression of the *MITF, tyrosinase*, and *TRP-2* in B16F10 cells treated with the fermented maca root extracts was confirmed. As shown in Figure 10, a 10% concentration of fermented maca root extracts considerably reduced the *MITF, tyrosinase*, and *TRP-2* mRNA expression compared to the same concentration of non-fermented maca root extracts. These results indicate that the fermented maca root extracts suppress melanin synthesis by inhibiting the expression of these three genes, potentially resulting in whitened skin.

## 4. Discussion

Maca root (*Lepidium meyenii*) is well known to have several physiologically active properties, including antioxidant, immunomodulatory, and anticancer effects, but there have been fewer studies on the effect of maca root on the skin [40]. However, maca root contains various polysaccharides, amino acids, phenolics, and minerals, implying that it is suitable as a raw material for cosmetic products [65]. Therefore, in this study, we tried to verify the skin-whitening effect of maca root. Especially, fermented plant extracts have better antioxidant, anti-melanogenic, anti-inflammatory, and anti-wrinkle effects compared with non-fermented plant extracts [66,67,68,69]. Fermented red ginseng showed whitening and anti-wrinkle effects [70], whereas fermented *Fructus acrtii* extracts suppressed the expression of MMP-1 in human fibroblasts (HDFs), and fermented hydroponic ginseng showed improved antioxidant, anti-inflammatory, and anti-melanin production [67]. Furthermore, fermented unpolished black rice (*Oryza sativa* L.) and fermented Viola mandshurica inhibited melanogenesis in B16F10 [71,72], while fermented soybean extracts showed higher antioxidant and skin-whitening effects than those in a non-fermented condition [73]. Furthermore, fermented black ginseng showed anti-wrinkle effects on HDF [74]. This suggests that fermented plant extracts are more effective cosmetic ingredients than their non-fermented counterparts. Based on these studies, we fermented maca root using *Lactobacilli* to investigate its effects on inflammation and melanogenesis.

*Lactobacilli* cause changes in components by breaking down macromolecules or amino acids during fermentation to produce several metabolites, such as bacteriocins, vitamins, and exopolysaccharides [75]. In particular, among the component changes caused by plant fermentation, changes in phenolic compounds mainly cause changes in antioxidant, anti-inflammatory, or anti-melanogenic activity [76]. Phenolic compounds are found in various fruit and vegetables and may provide health benefits by reducing the risk of developing metabolic disorders [77]. The biological properties of phenolic compounds are diverse and include antioxidant, anti-inflammatory, or properties that inhibit enzymes involved in the treatment of human diseases such as hypertension, type 2 diabetes, skin hyperpigmentation, or Alzheimer’s disease [78]. Fermentation can cause either an increase or a decrease in phenolic compounds. For example, the fermentation of Cactus Cladodes with lactic acid produced flavonoid derivatives (isorhamnetin and kaempferol), resulting in the production of antioxidant and anti-inflammatory properties [79]. Additionally, compared to non-fermented hydroponic ginseng (HPG), the total phenolic and flavonoid content of HPG fermented by Leuconostoc mesenteroides increased, showing antioxidant activity and anti-inflammatory effects by inhibiting the NO production and the expression of inflammatory cytokines, such as tumor necrosis factor-α, interleukin (IL)-1β, and IL-6 [80]. In addition, the increase in polyphenols, flavonoids, and polysaccharides in fermented vine tea using saccharomyces cerevisiae showed a strong scavenging ability for reactive oxygen species (ROS). Furthermore, it inhibited the intracellular tyrosinase activity and melanin synthesis in B16F10 and reduced inflammatory cell recruitment in a zebrafish [81]. Additionally, a further study has shown that cocoa seeds fermented with both yeast and LAB decreased in total polyphenol content (TPC) and antioxidant power [82]. These studies suggest that the increase or decrease in phenolic compounds during fermentation affects their antioxidant, anti-inflammatory, and anti-melanogenic effects. It is well known that maca root also contains phenolic compounds such as flavonoid, epigallocatechin gallate (EGCG), and epicatechin [65], and based on the above studies, we investigated the TPC of maca root extracts before and after fermentation and there was no significant difference in total polyphenol contents between the non-fermented and fermented maca root extracts at concentrations of 2.5, 5, and 10% (Table 2). Since these results were based on the total phenolic compound, further studies are needed to determine which phenolic-component changes during fermentation affect the anti-inflammatory and anti-melanin effects of fermented maca root extracts.

In addition to phenolic compounds, maca root is composed of several components such as amino acids, polysaccharides, and secondary metabolites [65]. Leucine, arginine, and glutamic acid account for a high proportion of the amino acids in maca root, and MC-1 and MC-2 are well known polysaccharides isolated from maca root [83,84]. In addition, macaenes and macamides are well-known secondary metabolites of maca root that exhibit antioxidant activity [38]. Numerous amino acids participate directly or indirectly in melanogenesis, and it is known that leucine and glutamic acid affect anti-melanogenic effects [85]. Leucine is important for tyrosinase inhibitory activity and glutamic acid is one of the constituent amino acids of glutathione which inhibits tyrosinase enzyme and converts eumelanin to pheomelanin production, which has a skin-whitening effect [85,86]. Additionally, arginine is known as a precursor to the synthesis of NO, an inflammatory mediator [87], and MC-1 and MC-2 exhibited immunomodulatory activity increasing the levels of NO, TNF-a and IL-6 in RAW 264.7 cells [42,88]. Figure 7 and Figure 9 show that the non-fermented and fermented maca root extracts dose dependently inhibited melanin production and intracellular tyrosinase activity in B16F10 cells. In addition, as shown in Figure 4, the non-fermented maca root extracts increased NO secretion in macrophages in a dose-dependent manner, and the fermented maca root extracts inhibited NO secretion compared to the non-fermented group at the same concentration. These results suggested that the fermentation using Lactobacilli increased their anti-melanogenic and anti-inflammatory effects. Based on the studies showing that Lactobacilli can use and produce amino acids [75,89,90] and polysaccharides [91,92], and the fact that maca root consists of various components, our results imply that during fermentation, Lactobacilli may change the components contained in maca root, causing a reduction in NO in macrophages and melanin in melanocytes. Therefore, further studies are needed to study which components of maca root extract change through fermentation.

Postbiotics can be obtained specifically through a fermentation process, meaning they contain many different types of metabolites. These metabolites confer various bioactive properties to postbiotics. Most metabolites with a physiological activity are produced by LAB and show various health benefit activities, such as antioxidant, anti-inflammatory, immunomodulatory, and anti-microbial activities [19,20,21,23,25]. Additionally, compared to probiotics, postbiotics show similar beneficial effects and higher stability and safety, meaning postbiotics are highly sustainable ingredients in cosmetic products [17]. In particular, previous studies have clinically proven that postbiotics can improve skin health. For instance, the oral intake of heat-killed cells of *Lactococcus lactis strain H61* improves women’s skin health [93]. In clinical trials, postbiotics of TAC/collagen have been shown to improve the skin’s moisture score and inflammatory index in vivo and accelerate the wound healing of acne symptoms in acne-prone volunteers. Additionally, this TAC/collagen gel reduced the number of brown spots and porphyrins on the facial skin [94]. Topical application of an anti-acne lotion containing a fermented lysate produced by *Lactiplantibacillus plantarum* has been shown to improve moisturization and regulate skin pH, with positive results in the treatment of acne [95]. Additionally, various products using postbiotics as ingredients exist in the cosmetic market [17]. Therefore, fermented maca root extract is highly likely to be applied as a cosmetic ingredient, and additional safety and clinical-efficacy tests are required.

The skin protects itself from solar irradiation through melanogenesis. When the skin is exposed to UV light, keratinocytes increase the transcription of the pro-opiomelanocortin gene in response to cellular damage. Pro-opiomelanocortin is a precursor protein that is cleaved to form α-MSH [96]. α-MSH binds to MC1R located on the surface of melanocytes as a factor that regulates melanocytes and skin pigmentation. The binding activates several pathways. The PKA-CREB and mitogen-activated protein kinase (MAPK) pathways are primarily involved in melanin production [63,97]. In melanogenesis associated with PKA-CREB signaling, α-MSH binding elevates intracellular adenylate cyclase, which increases cAMP concentration and PKA cell-signaling. Moreover, PKA phosphorylates the cAMP response element (CREB), which acts as a transcription factor for several genes, including the *MITF* [98,99]. The MAPK family comprises three types of protein kinases: extracellular reactive kinases (ERKs), c-Jun N-terminal kinases (JNKs), and p38 MAPKs [97]. The JUN MAPK and p38 MAPK pathways can stimulate melanogenesis by upregulating the *MITF* expression, and the ERK MAPK-dependent *MITF* expression pathway is involved in melanogenesis [100,101]. *Tyrosinase, TRP-1*, and *TRP-2* are the three main enzymes required for melanogenesis. The minor-eye-associated transcription factor (MITF), the master gene for melanocyte differentiation, regulates these three enzymes [102]. B16F10 cells treated with both fermented maca root extracts and α-MSH showed a suppressed *MITF, tyrosinase*, and *TRP-2* mRNA expression compared to those treated with only α-MSH, or both α-MSH and the non-fermented maca root extracts. These results show that the fermented maca root extracts improved anti-melanogenesis by inhibiting the expression of the *MITF, tyrosinase*, and *TRP-2*. Therefore, maca root extracts fermented using *Lactobacillus* have the potential to be used as a raw material in the production of cosmetic products. However, further research is needed to determine which ingredients and pathways exhibit the beneficial effects of these fermented plant extracts.

## 5. Conclusions

This study identified the antioxidative, anti-inflammatory, and anti-melanogenic effects of fermented maca root extract using *Lactobacilli*. Fermented maca root extracts maintained the antioxidant ability of the non-fermented maca root extracts and considerably reduced the production of NO, thereby demonstrating anti-inflammatory effects. In addition, fermented maca root extracts inhibited melanogenesis by inhibiting the expression and tyrosinase activity of the *MITF*, *tyrosinase*, and *TRP-2* mRNA. This shows that fermentation of maca root using *Lactobacillus* maintained its antioxidant effects and improved its anti-inflammatory and anti-melanogenic effects compared to the non-fermented maca root extracts. Postbiotics are currently in the limelight in the cosmetics industry for their safety, stability, and functional properties, and clinical trial research results on postbiotics as cosmetic ingredients are accumulating. Based on the results for fermented maca root extracts used in this study, clinical trials and cosmetic-product development using these postbiotics can be expected. Furthermore, the application of this postbiotic in minimally invasive procedures can be expected.

## Figures and Tables

**Table 1:** Primer sequences for RT-PCR.

Gene Name	Sequence	Base Pairs
mGAPDH [57]	F: 5′—ACATCAAGAAGGTGGTGAAG—3′ R: 5′—ATTCAAGAGAGTAGGGAGGG—3′	392 bp
mMITF [58]	F: 5′—AGCGTGTATTTTCCCCACAG—3′ R: 5′—TAGCTCCTTAATGCGGTCGT—3′	124 bp
mTyrosinase	F: 5′—CAGGCTCCCATCTTCAGCAGAT—3′ R: 5′—ATCCCTGTGAGTGGACTGGCAA—3′	132 bp
mTRP-2	F: 5′—GCAAGATTGCCTGTCTCTCCAG—3′ R: 5′—CTTGAGAGTCCAGTGTTCCGTC—3′	119 bp

**Table 2:** Total phenolic contents of the non-fermented and fermented maca root extracts.

Treatment	Total Phenolic Content (mg GAE/100 µL Extracts)		
	2.5% of Maca Root	5% of Maca Root	10% of Maca Root
Non-fermented	3.06 ± 0.02	4.91 ± 0.04	9.19 ± 0.01
*L. plantarum* KCTC 3108	3.01 ± 0.06	5.06 ± 0.06	8.93 ± 0.16
*L. rhamnosus* KCTC 5033	3.14 ± 0.10	5.05 ± 0.09	8.95 ± 0.02
*L. casei* KCTC 3109	2.94 ± 0.05	4.95 ± 0.08	8.97 ± 0.05
*L. gasseri* KCTC 3143	3.1 ± 00.09	5.18 ± 0.06	8.91 ± 0.16

Data are the results of three independent experiments.

## Data Availability

Data are contained within the article.
